# News Items—Medical Facts, &c.

**Published:** 1858-11

**Authors:** 


From the London Lancet.

*News Items, Medical Facts, &c.*

American Coffee and English Beer.—In the United States the Consumption of coffee is eight times as great as in Great Britain, and probably the consumption of beer in Great Britain is eight times as great as in the United States.

---

Death from Chloroform.—The "Northampton Herald" contains an account of a boy who died from the inhalation of chloroform sprinkled on a handkerchief. He had received an injury of the toe, and the chloroform was administered, in consequence of his excitement, to keep him quiet.

---

Sanitary Rules for Camps.—At one of the last meetings of the Academy of Sciences, Dr. Jules Cloquet gave an account of the Report on the Sanitary Condition of the Camp at Chalons, by Barron Larrey. This important document has attracted a large share of public attention. The author, who was specially appointed to attend his Majesty at the camp at Chalons in August, 1857, gives a detailed account of the sanitary condition of the troops assembled there at that period; and lays down rules, founded upon experience and sound reasoning, for the formation of camps, their internal police, and every element requisite for maintaining the health of an army in the field.

---

Medical Evidence.—Medical evidence should be in simple language when given before a jury. Really eminent men do not indulge in absurd technicalities, which are perfectly unintelligible to the community. The accompanying is a specimen from another class of witnesses. During a case of assault heard before judge Falconer, the following occurred: Surgeon examined—"I found plaintiff had a severe contusion under the left eye, great extravasation of blood under the eye, and some abrasion of the skin." Judge—"You mean that he had a bad black eye." Surgeon—"Yes."

---

The Legion of Honour and the Medical Profession.—No less than twelve crosses were lately given to medical men, both in civil and military practice. Amongst the higher grades of the order we notice Messrs. Andral and Trousseau. These eminent physicians have attained the rank of commander, which is the highest but one, in the Legion of honour.

---

Ricord and Chomel.—The late lamented Chomel was in the habit of retiring, at the end of each week, to a beautiful country seat which he had bought in the neighborhood of Paris. This fine estate was lately put to the hammer; and we are glad to find that it was knocked down to another illustrious member of our profession. M. Ricord is the purchaser, and thus will Chomel's chateau remain in professional hands. A fete was lately given at this country-seat by the syphilographist, who was warmly congratulated by his friends on having at last acquired some of the characteristics of the bee, besides those of zeal and perseverance in labor.

---

A Lucknow Surgeon.—The Pera arrived at Southampton on Saturday, bringing home from Calcutta, amongst other gallant and distinguished passengers, Dr. Partridge, 2nd Oude Irregular Cavalry. It will be remembered that this regiment mutinied in June, and killed three of their officers, viz., Lieutenants Barber and Fayer, and Captain Hays, the military secretary to the Chief Commissioner, Dr. Partridge was after this in Lucknow during the whole seige, in charge of the staff and native hospitals, and went through all the hard work and sufferings of that memorable siege.

---

Pepsin Wine.—We find in "L'Union Medicale" that the following pepsin wine is extremely agreeable and efficacious:—Take of starchy pepsin, prepared according to Messrs. Covisart and Bourdault's formula, one drachm and a half; distilled water, six drachms; white wine (of Lunch), fifteen drachms; white sugar one ounce; spirit of wine (33°), three drachms. Mix until the sugar is quite dissolved, and filter. One tablespoonful of this wine contains about fifteen grains of pepsin, and may be given after every meal.

---

Courageous Conduct of Military Medical Men in India—We have much pleasure in making the following extract from a despatch of General Whitlock, commanding the Sangor Field Division, to the Resident at Hyderabad: "On the 19th inst., (April) in driving the enemy out of a very strong position covering the road to Bandah, I have the gratification of bringing under your notice the conspicuous gallantry displayed by the officers and squadron of the Hyderabad Contingent, under Captain M'Intyre, in a charge the irregular cavalry made on the enemy's guns under a most galling fire of grape and musketry, and in face of a most determined body of horsemen, who fought most boldy. . . . To Captain M'Intyre, Lieutenant Ryal, and Surgeon Bradley, I am much indepted for their gallant conduct on this occasion; Surgeon Bradley attended his wounded under a very heavy fire."

---

Liberality of the late Professor Chomel.—This eminent member of our profession has left to the Medical Benevo'eut Society of the Department of the Seine three per cent, stock, bearing an interest of £8 a year.

---

The Life of a Plant.—The last report published by the Horticultural Society of the Seine Inferieure, states that some vegetables which were growing at Herculaneum at the time when that city was buried beneath the lava of Vesuvius in 79, and only recently dug up, acquired renewed vegetation on coming into contact with air and light.—

This statement seems difficult to be believed, as the natural question arises how did they escape the effects of burning lava?

---

Poisonous Effects of the Leaves of the Yew-Tree.—That the leaves of the yew are of pernicious influence was known to the Greeks and Romans, and confirmed by later authorities. The yew-tree itself may cause vertigo, lethargy, and even drunkenness, by the narcotic exhalations which it emits. We now read, in the *Journal des Landes*, that three horses belonging to the squadron of cavalry stationed at Mont de Maison, died there suddenly a few days ago, and that on dissection, it was found that they had been eating plentifully of the leaves of some yew-trees: the coats of the stomach evinced marks of the deleterious effects produced by this food.

